# Massachusetts State Veterinary Medical Association

**Published:** 1895-03

**Authors:** John M. Parker

**Affiliations:** Secretary


### SOCIETY PROCEEDINGS.

### MASSACHUSETTS STATE VETERINARY MEDICAL ASSOCIATION.

THE regular monthly meeting of the Massachusetts Veterinary Association was held at 19 Boylston Place on Wednesday, December 26th, at 7.30 P. M. The members present were Drs. Bunker, Burr, Emerson, Labaw, Osgood, Parker, Winchester and Winslow, and several visitors. Dr. Burr occupied the chair. The minutes of last meeting having been read, Dr. Beckett moved that they be adopted as read, seconded by Dr. Labaw and carried.

The names of Drs. Walker, of Taunton, a graduate of University of Pennsylvania, and A, J. Sheldon, of 50 Village street, a graduate of the American Veterinary College, were received and laid on the table for a month. The name of Dr. Sheldon was reported on favorably by the members of the Executive Committee present, Drs. Winchester and Labaw. Dr. Walker's credentials had not been received.

The matter of appointing a committee on legislation then came up. Dr. Winchester opposed the formation of such a committee, as it could be of no practical benefit, and would tend to bring the Association more or less into politics. He thought the Association should take no active part in any political movement. We have no axe to grind and no favors to ask, and we should keep out of it.

Dr. Parker supported the formation of such a committee. It is not right, he contended, that the Association should be so entirely ignorant of the legislation that is going on. He recalled the incidents of the past year when legislation of the greatest importance to the profession was enacted by the Legislalure, and yet the Association was entirely ignorant of such proposed legislation. Such a state of things should not be allowed to continue, and he moved that such a committee should be appointed by the Association. This motion was seconded by Dr. Beckett. Dr. Bunker supported the appointment of the committee; he was distinctly in favor of it, and on being put to the meeting it was carried unanimously.

Nominations were made from the floor, and election by ballot. The committee was appointed after two ballots, with the following result: Dr. Howard, 8 votes; Dr. Bunker, 6 votes, and Dr. Osgood, 6 votes.

The Secretary then asked the members for papers, and Dr. Bunker volunteered a paper for next meeting, and Dr. Winslow volunteered for February.

The Secretary then suggested that an effort should be made to make the meetings more interesting. He thought it would be a good idea if the Chairman were to ask each member present to report verbally and in a conversational way any interesting cases or any matter of interest to the profession. The idea was adopted, and the Chairman made a beginning by calling on Dr. Winchester to give the Association the benefit of any cases of special interest he had had lately.

Dr. Winchester reported a case of lameness in the stifle-joint. The joint was much swollen and nodular to the feel; it had been more or less enlarged for upward of a year. He opened it and extracted a number of tumors (eight), which had the appearance of fibrous tissue; made a good recovery.

Dr. Labaw reported a case of traumatic pleurisy in a mare in foal. She had kicked against a fence, and a portion of the fence had broken off and pierced the wall of the chest between the thirteenth and fourteenth ribs, exposing the lung. The external wound healed nicely, and she seemed to make a good recovery, with little rise in temperature. During the third week, however, the chest began to fill, and she finally died in about three weeks after injury. The interesting point in the case was that she lived so long after the lung had been exposed. He also referred to a case of osteo-sarcoma in a horse treated with potassium iodine, and recovered in three months.

Dr. Beckett reported a case of pneumonia in which death resulted from heart disease. A post-mortem examination showed a deposit of lime salts on the auricles. They had completely lost their contractile power. The condition was confined to the auricles. There was slight local pericarditis and little effusion.

Dr. Bunker reported a case of a cow supposed to have died from tuberculosis. Post-mortem examination showed piece of hay-wire in the heart. He also reported an autopsy in a cow in which they had found a tumor six or seven inches in diameter attached to the inside of the rumen. Dr. Burr said he thought it was a papilloma, but he had not yet made a microscopical examination of it.

Dr. Osgood spoke about abscesses in liver being of frequent occurrence. They were not of tuberculous origin. He advocated investigation being made to determine their character.

Dr. Parker reported case of cystitis in a mare. She had been in this condition for some time. She urinated and strained frequently. Digital examination showed the mucous membrane of the bladder to be thickened and easily removed. He removed a piece three or four inches square. The stench from the membrane was very bad. The bladder was washed out with boric-acid solution, and boric was given internally. The mare made good recovery. The peculiarity of the case was that a man had been seen to go to the mare's stall and raise himself by standing on a bucket, place his arm in the mare's vagina, and take "satisfaction" in this way. On one occasion, at least, he introduced his penis into the mare, and the question arose as to whether such an act would cause this condition in the mare.

There being no further business the meeting adjourned.

JOHN M. PARKER,
Secretary.

